# Metabolic regulation in the senescence process of stem cells

**DOI:** 10.1093/stcltm/szaf041

**Published:** 2025-09-19

**Authors:** YingYing Wei, Bin Zhang, Qingli Bie, Baoyu He

**Affiliations:** Key Laboratory of Cell and Biomedical Technology of Shandong Province, Affiliated Hospital of Jining Medical University, Jining Medical University, Jining, Shandong 272000, China; Department of Laboratory Medicine, Affiliated Hospital of Jining Medical University, Jining Medical University, Jining, Shandong 272000, China; Key Laboratory of Cell and Biomedical Technology of Shandong Province, Affiliated Hospital of Jining Medical University, Jining Medical University, Jining, Shandong 272000, China; Department of Laboratory Medicine, Affiliated Hospital of Jining Medical University, Jining Medical University, Jining, Shandong 272000, China; Shandong Medicine and Health Key Laboratory of Molecular Diagnostics & Precision Medicine, Affiliated Hospital of Jining Medical University, Jining Medical University, Jining, Shandong 272000, China; Key Laboratory of Cell and Biomedical Technology of Shandong Province, Affiliated Hospital of Jining Medical University, Jining Medical University, Jining, Shandong 272000, China; Department of Laboratory Medicine, Affiliated Hospital of Jining Medical University, Jining Medical University, Jining, Shandong 272000, China; Shandong Medicine and Health Key Laboratory of Molecular Diagnostics & Precision Medicine, Affiliated Hospital of Jining Medical University, Jining Medical University, Jining, Shandong 272000, China; Key Laboratory of Cell and Biomedical Technology of Shandong Province, Affiliated Hospital of Jining Medical University, Jining Medical University, Jining, Shandong 272000, China; Department of Laboratory Medicine, Affiliated Hospital of Jining Medical University, Jining Medical University, Jining, Shandong 272000, China; Shandong Medicine and Health Key Laboratory of Molecular Diagnostics & Precision Medicine, Affiliated Hospital of Jining Medical University, Jining Medical University, Jining, Shandong 272000, China

**Keywords:** anti-aging interventions, metabolic reprogramming, metabolites, stem cell senescence, therapeutic targets

## Abstract

Background: Aging is an inevitable and complex biological process characterized by progressive cellular and functional deterioration, leading to increased disease susceptibility and mortality. Stem cells, endowed with unique self-renewal and multipotent differentiation capabilities, play a pivotal role in tissue homeostasis and regenerative processes. However, the aging process triggers stem cell senescence, manifested by diminished proliferative capacity and differentiation potential, ultimately compromising tissue regeneration and contributing to the pathogenesis of various age-related disorders, including neurodegeneration, cardiovascular diseases, and metabolic syndromes.Main Findings: Metabolic plasticity serves as a fundamental mechanism enabling stem cells to dynamically adapt their energy requirements during self-renewal and lineage commitment. Emerging evidence indicates that cellular metabolism extends beyond its conventional role in energy production, actively participating in the regulation of stem cell fate decisions. Notably, nutrient-sensitive metabolites constitute a sophisticated metabolism-epigenetic axis that integrates metabolic flux, signaling pathways, and epigenetic modifications to precisely orchestrate cellular behavior. This regulatory axis is indispensable for maintaining tissue homeostasis and facilitating regeneration, thereby positioning metabolic reprogramming as a promising therapeutic strategy for mitigating aging-associated decline.Conclusions: In conclusion, elucidating the intricate crosstalk between stem cell metabolism and the aging process unveils novel opportunities for developing innovative anti-aging interventions and enhancing tissue repair. Future investigations should focus on the precise manipulation of metabolic pathways to effectively counteract age-related functional deterioration and promote longevity.

## Graphical abstract

**Graphical Abstract:**
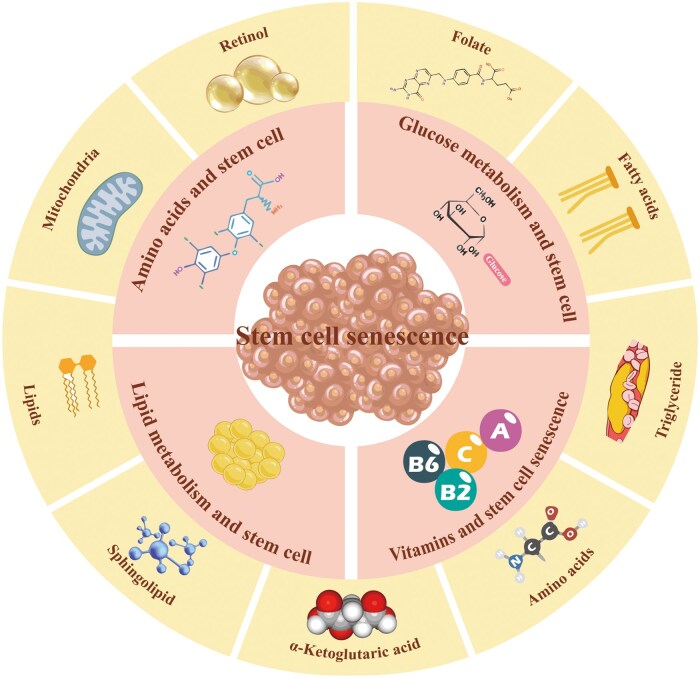

## Introduction

Aging is a complex, time-dependent biological process characterized by the gradual loss of physiological integrity, leading to functional decline in multiple tissues and organs.^1^ Cutting-edge research has demonstrated that the senescence of mesenchymal stem/stromal cells (MSCs) plays a pivotal role in the organismal aging process.^2^ As a major risk factor for various diseases, including cancer, diabetes, cardiovascular disorders, and neurodegenerative conditions, the progression of aging is closely linked to metabolic regulation.^3^ Research indicates that metabolic flexibility plays a central role in maintaining tissue homeostasis by modulating stem cell fate decisions—such as quiescence, proliferation, self-renewal, and differentiation—a process heavily dependent on energy substrates like glucose, fatty acids, and amino acids.^4^ Evidence supporting the connection between metabolism and aging is reflected in three key observations: (1) suppression of nutrient signaling extends lifespan, (2) enhanced anabolic signaling accelerates aging, and (3) pharmacological modulation of metabolic pathways effectively delays aging. Additionally, metabolic pathways can influence the rate of organismal aging by altering epigenetic states.^5^

Metabolites play multifaceted roles in regulating stem cell aging. Amino acid metabolism (eg, glutamine^6^ and NAD^+^ precursors^7^) maintains stem cell homeostasis by modulating mitochondrial function, antioxidant defense, and epigenetic modifications. Glucose metabolism reprogramming (eg, the balance between glycolysis and oxidative phosphorylation^8^) directly impacts stem cell energy supply and differentiation potential, while dysregulated lipid metabolism (eg, ceramide accumulation^9^) triggers oxidative stress and apoptosis, accelerating aging. Notably, small molecules such as vitamins (folate,^10^ ascorbic acid^11^) and metabolic intermediates (eg, α-ketoglutarate^12^) can delay stem cell senescence by targeting key pathways like mTOR, AMPK, and NRF2. A deeper understanding of these metabolic networks not only provides novel insights into the mechanisms of aging but also lays the theoretical foundation for developing intervention strategies, including metabolic reprogramming and small-molecule therapeutics, with significant implications for extending healthspan and combating age-related diseases.

## Association between amino acid and stem cells

Amino acids (AA) as the fundamental building blocks of proteins and various biomolecules play an indispensable role in maintaining physiological balance and metabolic homeostasis. As essential metabolites, amino acids participate in the biosynthesis of macromolecules such as nucleotides, glucosamine, and glutathione.^13^ As shown in Table 1, the regulatory role of amino acids and their related metabolites in stem cell aging. Amino acid metabolism serves multiple critical functions in stem cell aging, not only providing the basic materials for protein synthesis to ensure continuous renewal and replenishment of functional proteins for normal stem cell activities but also deeply engaging in cellular energy metabolism. When glucose and lipid energy supplies are insufficient, amino acids can enter the tricarboxylic acid (TCA) cycle through transamination and deamination reactions to provide energy for cells. Additionally, amino acid metabolism regulates stem cell aging comprehensively by modulating intracellular redox homeostasis and participating in signal transduction. Studies have shown that N-acetyl-L-cysteine (NAC) helps maintain the stemness of post-ovulatory oocytes *in vitro*, delaying their aging.^14^

**Table 1.** The regulatory role of amino acids and their related metabolites in stem cell senescence.

Metabolite	Biological process	Signaling pathways	Experimental model	Therapeutic potential
N-acetyl-L-cysteine^14^	Stemness; Antioxidative stress	N/A	Oocyte	Delay oocyte aging in assisted reproductive technology
Kynurenine^15^	Oxidative stress; lipid peroxidation	NF-κB; Nrf2	Bone; Muscle	Alleviate age-related bone loss and sarcopenia
β-Hydroxybutyric acid^16^	Oxidative stress; Homeostasis	PI3K/AKT/mTOR; Nrf2	Intestinal stem cells	Improve aging related intestinal dysfunction
Arginine^17^	Oxidative stress; Anti-inflammatory protection	SIRT1-AKT-Nrf2; SIRT1-FOXO3a	C2C12 Myotube cells; IPEC-J2 cells	Improve intestinal inflammation and metabolic diseases
L-β-Aminoisobutyric acid^18^	Anti-inflammation; Ferroptosis; ROS	Nrf-2	Osteocyte	Improve intestinal inflammation and metabolic diseases
Taurine^19^	Regeneration; Cell function	Nrf2; Wnt/β-catenin; BDNF-TrkB	Neural stem cells Skeletal muscle cells	Treatment of neurodegenerative diseases; Relieve Sarcopenia and age-related muscle atrophy
Selenomethionine^20^	Skin aging; Ferroptosis	NRF2/GPX4	Epidermal stem cells	Skin aging intervention and antioxidant therapy
Proline^21^	Mitochondrion	PINK1/Parkin	Human primary fibroblasts; IMR-90 cells	Delaying skin stem cell aging
Picolinic acid^22^	Differentiation; mineralization; bone density	IGF-1/PI3K/AKT	MC3T3-E1 osteoblast cell line	Delay bone aging
Poly-L-lysine^23^	Proliferation	FAK/PI3K/AKT	Mesenchymal Stem Cells	Avoid age-related functional loss
Pyruvate^24^	Autophagy; Cell differentiation	AMPK/mTOR	Human embryonic stem cells	Maintain stem cell homeostasis
Cystine^25^	Redox; Proliferation	NRF2; Wnt	Intestinal stem cell	Enhance small intestine proliferation; Delay intestinal aging
Leucine^26^	Protein synthesis	PI3K/AKT/mTOR	Skeletal muscle of elderly rats	Promote skeletal muscle regeneration
Serine^27^	Epigenetic regulation	GCN2	Dental pulp stem cells	Apply for pulp regeneration or tissue engineering
Glycine^28^	Epigenetic modification	GCS	Pluripotent stem cells	Delay stem cell aging
Threonine^29^	Stemness	N/A	Mouse embryonic stem cells	Delay the tissue stem cells aging
Glutamine^30^	Ferroptosis; Plasticity; Antioxidant defense	TCA; PI3K/AKT/mTOR; Nrf2	Muscle stem cells; Hair follicle stem cells	Inhibit iron death; Sarcopenia or age-related muscle atrophy; Treating age-related hair loss
S-adenosylmethionine^31^	Heterochromatic	PI3K/AKT/FOXO3a	Muscle stem cell	Treating muscle atrophy and regenerative disorders
Asparagine^32^	Function	PI3K/AKT/mTOR; Autophagy lysosome pathway	Intestinal stem cells; Hair follicle stem cells	Delay intestinal aging

### Amino acids and oxidative stress

Studies have shown that kynurenine (KYN) significantly affects bone metabolism in 12-month-old mature mice, leading to reduced bone mass, enhanced osteoclast activity, increased bone marrow fat content, and suppressed osteoblast activity.^15^ This observation aligns with recent findings elucidating the role of tryptophan metabolic dysregulation in skeletal aging. Specifically, the study demonstrates that kynurenine (KYN) promotes adipogenic over osteogenic differentiation of bone marrow mesenchymal stem cells (BMSCs) through activation of the aryl hydrocarbon receptor (AHR) signaling pathway, thereby exacerbating osteoporotic pathogenesis.^33^ Elevated levels of KYN may induce oxidative stress, triggering muscle atrophy, and lipid peroxidation, ultimately contributing to sarcopenia.^34^ The investigation further demonstrates that KYN suppresses the proliferative potential of muscle stem cells (MuSCs) while concomitantly enhancing proteolytic activity through upregulation of key muscle atrophy markers, including Atrogin-1 and MuRF1,^33^ This provides novel molecular insights into the dual role of KYN in sarcopenia pathogenesis by simultaneously affecting both the skeletal and muscular systems.

Atrazine exposure induces oxidative damage and premature senescence in hypothalamic neural stem cells (htNSCs) by hyperactivating the integrated stress response (ISR) pathway, particularly the PERK/eIF2α/ATF4 axis. In contrast, metabolites such as β-hydroxybutyrate (β-HB) may help maintain stem cell homeostasis by antagonizing ISR signaling.^16^ In the liver, β-hydroxybutyrate (β-HB) is produced through lipolysis and exhibits significant anti-aging and antioxidant effects in Drosophila models. Studies show that β-HB effectively inhibits age-related and oxidative-stress-induced centrosome amplification, regulates stem cell overproliferation, reduces DNA damage accumulation, and maintains heterochromatin stability in intestinal stem cells (ISCs) and their niche cells (ECs). These findings suggest that β-HB has the potential to maintain stem cell homeostasis through dual mechanisms.^35^ Notably, short-term supplementation of β-hydroxy-β-methylbutyrate (HMB) in aged mouse models significantly preserves muscle strength,^36^ further supporting the anti-aging and physiological function-maintaining effects of β-hydroxybutyrate derivatives. The high concentrations of glutamate promote the survival and proliferation of adult rat neural stem/progenitor cells (NSPCs) through non-N-methyl-D-aspartate (non-NMDA) ionotropic glutamate receptor-mediated signaling pathways. Glutamate also alleviates oxidative stress damage in NSPCs during *in vitro* culture by activating AMPA/kainate receptors.^37^ Moreover, L-arginine demonstrates multiple protective effects: it enhances *Caenorhabditis elegans* tolerance to oxidative and heat stress, extending lifespan,^17^ and protects against oxidative stress and inflammatory responses induced by lipopolysaccharide in myotubes and porcine intestinal epithelial cells.^38^^,^^39^ These findings provide new insights into the regulatory mechanisms of amino acid metabolites in cellular aging and stress responses.

### Amino acids and ferroptosis

As essential substrates for life activities, amino acids play a key role in regulating ferroptosis. Studies indicate that amino acid metabolism participates in the ferroptosis regulation process by maintaining iron homeostasis, lipid balance, and redox status.^40^ Ferroptosis is an iron-dependent form of programmed cell death characterized by the accumulation of lipid peroxides, with its pathological basis rooted in damage to the antioxidant system.^41^ Notably, the accumulation of senescent cells in bone tissue promotes ferroptosis *via* SASP (senescence-associated secretory phenotype) secretion. Clearance of these senescent cells has been shown to ameliorate bone degenerative diseases, such as osteoporosis.^42^ Recent research has identified several amino acids and their metabolites as critical regulators of ferroptosis. Extracellular L-glutamine and transferrin are important ferroptosis regulators, while intracellular glutaminolysis and transferrin transport-related components are necessary for ferroptosis occurrence.^43^ L-β-aminoisobutyric acid (L-BAIBA), a natural metabolite of valine,^18^ possesses anti-inflammatory and fatty acid oxidation-inducing properties.^44^ It also inhibits ferroptosis *via* the Nrf-2 signaling pathway,^45^ protecting cells from ROS-induced damage.^46^ Taurine, a sulfur-containing amino acid metabolite, counteracts Erastin-induced ferroptosis by stabilizing the iron pool and improving redox homeostasis.^19^ In skin aging, arachidonic acid (AA) accumulation leads to lipid peroxidation and mitochondrial damage in GPX4-deficient epidermal stem cells, triggering ferroptosis. Selenomethionine (Se-Met) protects cells from AA-induced damage by promoting GPX4 expression and enhancing antioxidant capacity.^20^ Additionally, selenomethionine alleviates oxidative stress and ferroptosis induced by BDE209 through the NRF2/GPX4 pathway.^47^ These studies demonstrate that amino acids and their metabolites regulate ferroptosis through multiple mechanisms, playing a pivotal role in cellular protection.

### Amino acids regulating mitochondrial function

Research has shown that mitochondrial transfer can enhance bone marrow mesenchymal stem cell function *in vitro* and promote *in situ* bone defect repair by upregulating aerobic metabolism, representing a promising new technique for optimizing stem cell therapy. Taurine modulates neural stem cell (NSC) proliferation signaling pathways by regulating mitochondrial function, providing a favorable microenvironment for NSC proliferation and differentiation.^48^ Similarly, as a mitochondrial stabilizer, *MAVS* counteracts senescence in human stem cells by preserving mitochondrial membrane potential and reducing reactive oxygen species (ROS) accumulation, further underscoring the critical role of mitochondrial quality control in stem cell function.^49^ Nicotinamide adenine dinucleotide (NAD^+^), a redox-active metabolite, is depleted in aging rodents, contributing to degenerative diseases. NAD^+^ metabolites are limited in aging cells due to reduced mitochondrial function, and their supplementation restores mitochondrial and stem cell function in various tissues while extending lifespan in mice.^7^ NAD^+^ precursor nicotinamide riboside (NR) treatment induces the mitochondrial unfolded protein response and suppresses protein synthesis, rejuvenating muscle stem cells (MuSCs) in aged mice.^50^ Blood analysis helps identify NAD^+^ deficiency, and studies suggest niacin as an effective NAD^+^-boosting therapy for mitochondrial myopathy.^51^ Mitochondrial modulator urolithin A corrects mitochondrial function in hematopoietic stem cells (HSCs), fully restoring the blood reconstitution capacity of aged HSCs. Urolithin A supplementation also restores the lymphocyte compartment, enhances HSC function, and improves immune responses to viral infections in aged mice.^52^ Aged stem cells exhibit defective mitophagy, leading to impaired mitochondrial function and depolarized mitochondria accumulation. Proline reduces ROS and induces mitophagy, clearing dysfunctional mitochondria and restoring mitochondrial function to reverse multiple aging markers.^21^ These findings highlight the significance of mitochondrial function regulation in stem cell therapy and anti-aging research, with methods such as mitochondrial transfer, NAD^+^ supplementation, and mitophagy induction effectively restoring stem cell function and delaying aging.

### Amino acids and cell proliferation/differentiation

Tryptophan and its metabolites play a crucial role in regulating stem cell properties and differentiation. Studies have shown that tryptophan and tyrosine enhance the stemness of bone marrow mesenchymal stem cells (BMSCs), increasing stem cell marker expression and self-renewal capacity while promoting osteogenic differentiation and upregulating osteogenesis-related genes. L-tryptophan and L-kynurenine enhance BMSC stem cell phenotypes, contributing to bone homeostasis maintenance.^53^ Additionally, tryptophan catabolite picolinic acid (PIC) induces osteogenic differentiation of mesenchymal stem cells *in vitro*.^22^ Poly-L-lysine (PLL)-coated culture systems increase MSC proliferation rates and alter gene expression related to stemness and differentiation potential, even reversing aged MSCs.^23^

Amino acid metabolites have multifaceted roles in stem cell regulation. Pyruvate, a key metabolite, influences human embryonic stem cell (HESC) differentiation by modulating metabolic balance and kinase cascades, particularly through AMPK activation and mTOR inhibition to enhance mesodermal differentiation.^24^ Cystine restriction enhances Wnt signaling, affecting intestinal stem cell marker expression and proliferation.^25^ Nutritional interventions like leucine supplementation improve skeletal muscle regeneration in aged rats by regulating the PI3K/Akt/mTOR pathway and ubiquitin-proteasome system.^26^ Mesenchymal stem cell and NSC proliferation and differentiation depend on balanced fatty acid oxidation.^54^ Hsf1 activation promotes HSC self-renewal and proliferation.^55^ Elevated N-acetylcysteine (NAC) antioxidant levels enhance cardiomyocyte regeneration, while reduced NAD^+^ levels affect histone acetylation, inducing myogenic programs during muscle stem cell activation.^56^ Aging reduces dental pulp stem cell (DPSC) proliferation and osteogenic differentiation, linked to weakened serine metabolism and p16 hypomethylation, offering new directions for addressing aging-related phenotypes.^27^

### Amino acids regulating cellular physiological functions

Amino acids play a vital role in regulating cellular physiological functions through multiple mechanisms. In stem cell fate regulation, the glycine cleavage system (GCS) determines pluripotent stem cell (PSC) fate by modulating aging-related pathways and epigenetic modifications. GCS is highly active in PSCs, with its rate-limiting enzyme Gldc regulated by *Sox2* and *Lin28A*. Activated GCS maintains stem cell pluripotency by promoting H3K4me3 modifications and suppressing cellular senescence.^28^

Metabolically, embryonic stem cells (ESCs) require threonine to support anabolic pathways like purine synthesis. Threonine deficiency impairs cell growth and depletes stem cell markers.^29^ α-KG and glutamine levels decline in aged mice, and their supplementation improves muscle regeneration in Psat1 conditional knockout and aged mice, suggesting therapeutic potential.^30^

Epigenetically, reduced heterochromatin in aged muscle stem cells (MuSCs) correlates with depleted methyl donor S-adenosylmethionine (SAM). Restoring SAM levels increases heterochromatin markers, alleviating age-related DNA damage, cell death, and impaired muscle regeneration. Excessive SAM consumption during polyamine synthesis reduces its availability for methylation, and inhibiting polyamine synthesis restores SAM levels and heterochromatin formation, improving aged MuSC function and regeneration. This reveals a direct causal link between polyamine metabolism and epigenetic dysregulation in MuSC aging.^31^ S-adenosyl-L-methionine (SAM) exhibits anti-aging effects *via* the PI3K/AKT/FOXO3a axis, offering new insights into MSC aging dynamics.^57^ Interestingly, SAM biosynthesis increases during Drosophila ovarian aging, linked to elevated Sam-S levels in germline cells.^58^

In post-translational modification regulation, the mechanisms of arginine methylation in MSC fate remain unclear. Further research on arginine methylation and other modifications, combined with protein arginine methyltransferase (PRMT) inhibitor development, may yield new therapies for sarcopenia and Duchenne muscular dystrophy (DMD).^59^

In combination therapies, metformin (MET) and leucine (LEU) co-treatment significantly benefits aged mouse muscle during disuse, addressing strength loss (independent of muscle size) and increasing satellite cell content while promoting collagen remodeling during recovery. MET+LEU enriches myogenesis-related transcriptional pathways and reduces inflammation, synergistically improving aged muscle quality.^60^

In intestinal stem cell regulation, asparagine (Asn) enhances aged Drosophila ISC function by activating the autophagy-lysosome pathway, suppressing intestinal hyperplasia and barrier damage, and mitigating age-related intestinal decline.^32^ In hair follicle stem cell regulation, mTORC2-Akt signaling inhibits glutamine metabolism during the hair cycle’s telogen phase, returning progenitors to hypoxic niches and restoring stemness for long-term maintenance.^6^ In neuroregeneration, taurine exhibits neuroprotective effects in aged mice, stimulating multiple aspects of adult neurogenesis and modulating microglial function *via* direct and indirect mechanisms.^61^ These findings deepen our understanding of amino acid roles in cellular physiology and offer new strategies for aging-related diseases.

## Association between glucose metabolism and stem cell senescence

α-Ketoglutarate (AKG), a key TCA cycle metabolite, plays a central role in cellular energy metabolism, amino acid/protein synthesis, epigenetic regulation, stemness, and reproductive health.^62^ Under normal conditions, stem cells flexibly adjust glucose metabolism to maintain functionality and stemness. As detailed in Table 2, glucose-related metabolites exhibit specific regulatory patterns in stem cell aging processes. However, aging or pathological factors disrupt glucose metabolism, impairing stem cell function.^8^

**Table 2.** The regulatory role of glucose-related metabolites in the senescence of stem cells.

Metabolite	Biological process	Signaling pathways	Experimental model	Therapeutic potential
α-ketoglutaric acid^8^	Epigenetic modification; ROS	TCA; PI3K/AKT/mTOR	Embryonic stem cells; Periodontal ligament stem cells; Mesenchymal stromal precursor cells	Promote collagen synthesis and reduces wrinkles; protect neurons; enhance osteoblast differentiation
3-phosphoglycerate^30^	Proliferation	Wnt/β-catenin	Muscle stem cells	Combat age-related muscle atrophy
Succinic acid^63^	Differentiation	Wnt/β-catenin; PI3K/AKT/mTOR	Embryonal stem cell	Inhibite embryonic stem cells differentiation

AKG regulates cellular aging through multiple mechanisms: inhibiting mTOR signaling and ATP synthase activity; modulating DNA and histone demethylation to influence epigenetic modifications; and reducing ROS to alleviate oxidative stress.^12^ Metabolic interventions like caloric restriction, intermittent fasting, exercise, and ketogenic diets modulate the TCA cycle to delay aging but have limitations.^64^

In differentiation regulation, persistent oxidative metabolism in mitochondrial-dysfunctional or aged stromal precursor cells causes differentiation failure. In Sod2-deficient cells, elevated mitochondrial ROS triggers metabolic reprogramming, leading to AKG overaccumulation, which induces cell death via enhanced DNA damage, Hif-1α destabilization, and reduced H3K27 acetylation.^65^ Similarly, TGF-β signaling-driven senescence in periodontal ligament stem cells (PDLSCs) manifests as an imbalanced α-KG/succinate ratio and decreased H3K27ac levels, further supporting the conservation of the metabolism-epigenetics axis in stem cell senescence across tissues.^66^ The intracellular αKG/succinate ratio regulates embryonic stem cell differentiation, indicating chromatin responsiveness to metabolic changes.^63^

In hematopoiesis, proteomic analyses reveal elevated glycolysis as a hallmark of aged hematopoietic stem/progenitor cells (HSPCs), suggesting interventions targeting central carbon metabolism or mTORC1 signaling for senescent cell clearance.^67^

During muscle regeneration, the serine biosynthesis pathway (SBP) initiating from glycolytic intermediate 3-phosphoglycerate (3-PG) is specifically activated. Psat1-generated α-KG and glutamine regulate MuSC activation and myogenic progenitor expansion,^30^ offering new metabolic perspectives for muscle regeneration.

## Association between lipid metabolism and stem cell senescence

Lipid metabolism plays a critical role in stem cell senescence regulation, involving fatty acid synthesis, transport, oxidation, and lipid signaling molecules, closely linked to stem cell function and fate.^8^  Table 3 systematically categorizes the regulatory functions of lipid metabolites in stem cell aging.

**Table 3.** The regulatory role of lipid-related metabolites in the senescence of stem cells.

Metabolite	Biological process	Signaling pathways	Experimental model	Therapeutic potential
Lipid aldehyde^68^	Carbonyl stress; Mitochondrial damage	PINK1/Parkin; cGAS-STING	IMR90 fibroblasts; Mouse adipose derived stem cells	Target products for AD and fatty liver
Ceramides^69^	Differentiation; Regeneration;	AKT/mTOR; NF-κB/STAT3	Bone marrow mesenchymal stem cells (BMSCs)	Target ceramide for skin stem cell aging
Fatty acid^70^	Metabolic reprogramming	TCA	Adult neural stem cells	Delay age-related cognitive decline or neurogenic disorders in Alzheimer’s disease
Bile acid^71^	Regeneration; Proliferation; Differentiation	cAMP-PKA-CREB; Wnt/β-catenin	Intestinal stem cells	Treat age-related intestinal dysfunction
Sphingolipids^72^	Cell apoptosis; Inflammation; Metabolic homeostasis	PI3K/AKT	Muscle stem cells; Human fibroblasts	Reduce systemic side effects

Lipid metabolites play a complex yet crucial regulatory role in stem cell aging processes. Multiple studies demonstrate that lipid peroxidation products such as 4-hydroxynonenal (4-HNE) can directly activate cellular senescence pathways by inducing carbonyl stress and mitochondrial dysfunction, leading to significant declines in stem cell proliferative capacity.^68^ Notably, ceramides exhibit a remarkable dual effect: while age-accumulated C24:1 ceramide promotes mesenchymal stem cell senescence through extracellular vesicle transmission,^69^ it conversely maintains stemness in intestinal stem cells by enhancing fatty acid oxidation.^70^ This seemingly paradoxical phenomenon suggests that lipid metabolites influence stem cells in a highly concentration-dependent and tissue-specific manner.

Various lipid metabolites participate in stem cell aging regulation through distinct mechanisms. Kukreti and Amuthavalli^9^ found that miR-34a-mediated inhibition of ceramide kinase (CERK) in skeletal muscle stem cells leads to ceramide accumulation, thereby impairing insulin signaling pathways. In neural stem cells, fatty acid oxidative metabolic switching has been identified as a key mechanism maintaining their activity.^54^

Additionally, bile acids activate intestinal stem cells and promote epithelial regeneration through TGR5 receptors,^71^ demonstrating the important role of lipid molecules in regulating stem cell microenvironments. These findings collectively construct a sophisticated “lipid metabolism-stem cell function” regulatory network.

Building on current research, Li and Kim proposed the “sphingolipid balance hypothesis,” suggesting that sphingolipid molecules of varying concentrations and types play different roles in stem cell aging.^72^ Clemot et al. further indicated that lipid metabolites may determine stem cell fate by establishing specific metabolic signatures.^73^ These discoveries not only deepen our understanding of stem cell aging mechanisms but also provide theoretical foundations for developing anti-aging interventions targeting lipid metabolism.

## Association between vitamins and stem cell senescence

Vitamin compounds play a crucial role in regulating stem cell function and the aging process. As systematically categorized in Table 4, vitamins and their derivatives exhibit distinct regulatory patterns in stem cell aging. Studies have demonstrated that folic acid exerts critical regulatory effects on the proliferation and differentiation of C2C12 myoblasts, suggesting that vitamin B family members may influence stem cell differentiation potential and thereby participate in aging modulation.^10^ Furthermore, retinol—a vitamin A derivative—exhibits anti-skin-aging properties suggesting that vitamin A compounds may delay tissue aging by modulating skin stem cell function.^74^ Ascorbate has been demonstrated to directly regulate hematopoietic stem cell function and influence leukemogenesis, suggesting that vitamin C can maintain stem cell homeostasis through epigenetic modification and related pathways.^11^ Collectively, these studies demonstrate that vitamin compounds influence stem cell behavior through distinct molecular mechanisms, thereby exerting multifaceted regulatory effects on cellular aging and tissue regeneration.

**Table 4.** The regulatory role of vitamins in the senescence of stem cells.

Metabolite	Biological process	Signaling pathways	Experimental model	Therapeutic potential
Folic acid^10^	Proliferation; Differentiation; Epigenetic regulation	MyoD/MEF2; PI3K/AKT/mTOR	C2C12 myoblasts	Improve skeletal muscle development disorders and aging
Ascorbic acid^11^	Epigenetic reprogramming; antioxidant	N/A	Mouse hematopoietic stem cells	Leukemia prevention and hematopoietic function regulation
Retinol^74^	Epidermal renewal; Collagen synthesis; Antioxidant defense	NRF2	Skin stem cells	Delay skin aging

## Research summary

This study systematically reviews metabolic regulation in stem cell senescence, revealing it as a complex biological process involving multi-level coordination. The mechanistic basis by which amino acid, glucose, lipid, and vitamin metabolism coordinately regulate stem cell aging through specific signaling pathways is schematized in Figure 1. Stem cell senescence is characterized by declined self-renewal, differentiation potential, and metabolic imbalance, manifesting as reduced cell volume, organelle dysfunction, and limited proliferation/differentiation. Metabolic regulation serves as the “core hub,” precisely controlling stem cell fate through glucose, lipid, and amino acid pathways. Dysregulation in any pathway disrupts stem cell homeostasis, accelerating aging. Specifically, glucose metabolism disorders impair energy supply and cause metabolite accumulation; lipid abnormalities damage membranes and exacerbate oxidative stress; amino acid imbalances affect protein homeostasis and signaling—collectively forming the “metabolic basis” of stem cell aging.

**Figure:**
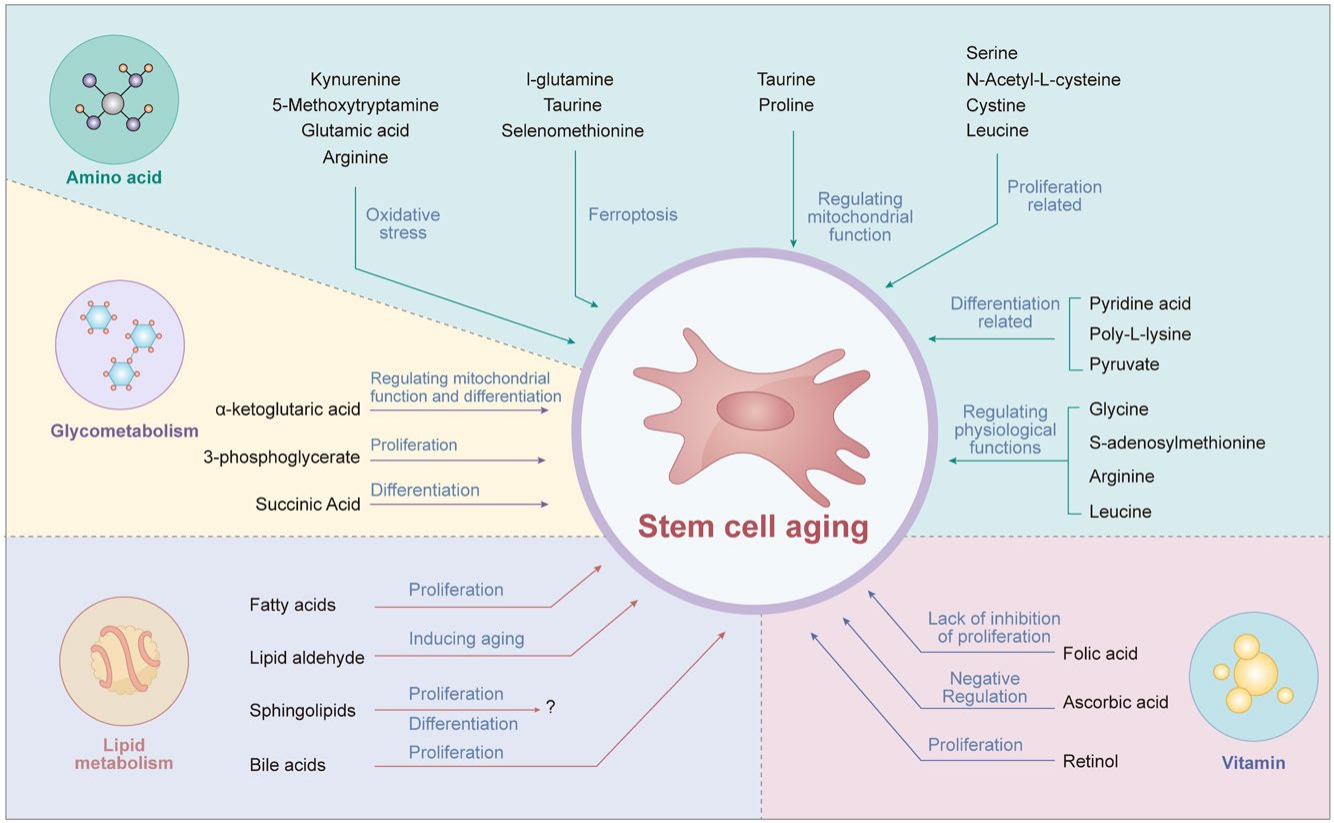
Figure 1. Regulatory roles of amino acids, glucose, lipids, vitamins and their metabolites in stem cell aging.

Metabolic regulators play “core roles” in stem cell aging. Transcription factors NRF2 and PGC-1α, as “key regulators” of antioxidant defense and mitochondrial function, precisely control gene expression to combat oxidative damage and maintain energy homeostasis. The mTOR and AMPK pathways act as “bidirectional switches”: mTOR promotes growth under nutrient-rich conditions but accelerates aging when overactivated, while AMPK senses energy stress to activate adaptive responses, counteracting mTOR to maintain metabolic balance. Intrinsic (genetic mutations, epigenetic changes) and extrinsic (nutrient deficiency, signaling disruption, oxidative stress, inflammation) factors jointly “shape” stem cell aging, ultimately causing metabolic defense system “collapse.”

Current metabolic-based interventions show promise in reversing stem cell senescence. Small molecules (eg, rapamycin, resveratrol) target key metabolic nodes; natural products (eg, curcumin, quercetin) exert antioxidant, anti-inflammatory, and metabolic effects. In gene therapy, CRISPR-Cas9 serves as a “gene-editing tool” for precise mutation repair, while engineered cell transplants act as “metabolic remodeling engines” to rebuild metabolic networks and reactivate tissue regeneration. These multi-dimensional strategies target critical aging mechanisms, laying foundations for combating aging-related diseases and extending healthspan.

## Future research directions

Despite progress in stem cell senescence research, its complexity demands further exploration. Future studies should construct detailed metabolic network maps to elucidate interactions between pathways, regulators, and environmental factors. Single-cell multi-omics can analyze metabolic, transcriptomic, proteomic, and epigenomic dynamics to identify cell-specific regulatory nodes. Systems biology modeling can simulate network dynamics and predict intervention outcomes, guiding targeted therapies.

The development of effective anti-aging interventions requires a multi-pronged approach targeting key metabolic pathways in stem cell senescence. For amino acid-based therapies, NAD+ precursors like nicotinamide riboside have shown promise in clinical trials (Phase III for age-related muscle decline), though their tissue-specific bioavailability remains challenging. Tryptophan metabolites, particularly KYN pathway modulators, demonstrate dual osteo-muscular effects but face hurdles in achieving targeted delivery to bone marrow and muscle stem cell niches. The differential effects of branched-chain amino acid metabolites (eg, L-BAIBA’s tissue-specific protection versus valine’s pro-aging effects) necessitate precise metabolic engineering approaches.

In lipid metabolism interventions, ceramide-modulating strategies illustrate the complexity of clinical translation—while C24:1 ceramide promotes MSC senescence, it enhances intestinal stem cell function, demanding tissue-specific delivery systems. Bile acid therapeutics targeting TGR5 receptors have advanced to Phase II trials for intestinal regeneration, yet systemic effects on other stem cell pools require further investigation. The “sphingolipid balance hypothesis” presents both opportunities and challenges for clinical application, as the therapeutic window for various sphingolipid species appears remarkably narrow in human studies.

Glucose metabolism modulators face distinct translational barriers. While α-ketoglutarate supplementation shows efficacy in preclinical muscle stem cell activation models, its rapid systemic clearance and dose-dependent effects on differentiation pose formulation challenges. Glycolysis inhibitors demonstrate potential in aged hematopoietic stem cells but risk compromising energy homeostasis in other tissues. The serine-glycine-one-carbon pathway emerges as a promising target, though its interconnectedness with folate metabolism requires careful clinical evaluation to avoid off-target epigenetic effects.

Current limitations across all metabolic approaches include: (1) inadequate biomarkers for stem cell-specific aging assessment, (2) insufficient understanding of age-related changes in drug metabolism, and (3) lack of standardized protocols for combinatorial therapies. The most advanced clinical candidates (NAD^+^ boosters, TGR5 agonists) still struggle with optimal dosing regimens and long-term safety profiles. Future development should prioritize: (1) engineered delivery systems for tissue-specific targeting, (2) metabolic profiling to identify patient subgroups, and (3) innovative trial designs incorporating functional stem cell readouts alongside traditional clinical endpoints. These efforts, coupled with strengthened academia-industry collaborations, could transform metabolic interventions from laboratory observations into viable clinical therapies for stem cell rejuvenation.

## Data Availability

No new data were generated or analyzed in support of this research.
